# Postoperative Intracranial Hemorrhage after an Endoscopic L5-S1 Laminectomy and Discectomy: A Case Report and Literature Review

**DOI:** 10.3390/jpm13020196

**Published:** 2023-01-22

**Authors:** Yizhou Xie, Xi Mei, Shanyu Liu, Brian Fiani, Xiaohong Fan, Yang Yu

**Affiliations:** 1 Hospital of Chengdu University of Traditional Chinese Medicine, No.39 Shi-er-qiao Road, Chengdu 610072, China; 2 Department of Neurological Surgery, Weill Cornell Medical College-New York Presbyterian, New York, NY 10065, USA

**Keywords:** postoperative intracranial hemorrhage, spine endoscopic surgery, leakage of CSF, constant irrigation, case report

## Abstract

Background: Postoperative intracranial hemorrhage (PIH) is a fairly rare but catastrophic perioperative complication following lumbar spine surgery. This is a case report of a 54-year-old male patient who experienced PIH 2 h after an endoscopic L5-S1 laminectomy and discectomy. Case Presentation: A 54-year-old male patient presented with right L5-S1 radiculopathy that corresponded with the picture revealed in medical imaging and the signs seen upon physical examination. Subsequently, he underwent endoscopic L5-S1 laminectomy and discectomy. The patient presented with idiopathic unconsciousness and limb twitching 2 h after surgery. An emergency cranial CT scan was obtained which demonstrated intracranial hemorrhage. Following an emergency consultation with the Department of Neurology and Neurosurgery, the patient underwent an emergency interventional thrombectomy as per their orders. The surgery was performed successfully. However, the patient’s situation did not improve and he died on the second postoperative day. Conclusion: PIH after spinal endoscopic surgery is a rare but horrible complication. Several factors could lead to PIH. However, in this patient, the cause of PIH might be attributed to the long operation time combined with cerebrospinal fluid (CSF) leakage. Great attention should be attached to the issue of PIH development in spinal endoscopic procedures due to constant irrigation. This study aims to highlight the issue of PIH following endoscopic spinal surgery by presenting a case report of a patient who died despite successful surgery.

## 1. Background

Postoperative intracranial hemorrhage (PIH) is a fairly rare but horrible complication that can follow endoscopic spinal surgery [1,2,3]. The incidence of PIH occurring after any spinal surgery is about 0.12% to 0.74%, as reported in the literature so far [4,5,6,7]. Although the cause remains controversial, most literature reports regard intracranial hypotension secondary to CSF leakage as a main factor [8,9,10]. However, a few other crucial elements might be ignored, especially in endoscopic spinal surgery. This is a case report of a 54-year-old male patient who died due to PIH after undergoing an endoscopic L5/S1 laminectomy and discectomy.

## 2. Case Presentation

### 2.1. Study Design

Case report.

### 2.2. Basic Information

**Figure 1:**
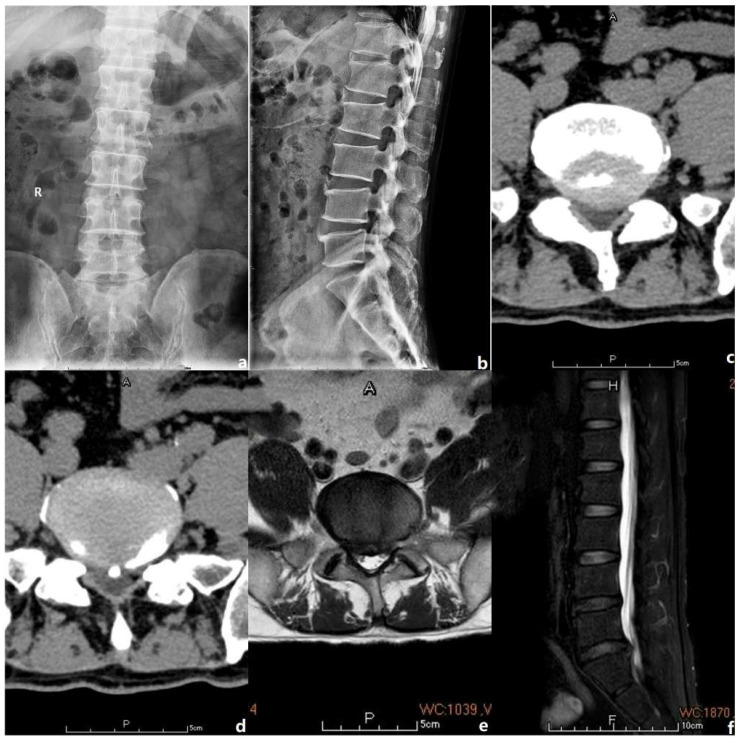
The preoperative images of the case. The letter R, A, P, F, H in the figures refers to Right, Anterior, Posterior, Foot and Head, respectively. (**a**,**b**) Preoperative anterior and posterior lumbar spine radiographic films. (**c**,**d**) Preoperative lumbar CT scan showed L5/S1 canal stenosis with calcified disc protrusion. (**e**,**f**) Preoperative lumbar MRI manifested L5/S1 canal stenosis with disc herniation.

Informed consent was obtained from the patient and his relative before starting the treatment. The study was reviewed by the ethics committee of the corresponding author’s affiliated institution (approval no. NT-7964). Informed consent was obtained from all candidates before their participation in the study. Hence, in this article, a 54-year-old male patient presenting with right L5-S1 radiculopathy that corresponded to the image revealed on medical imaging and signs observed upon physical examination is reported (Figure 1). After completing the preoperative laboratory examination, which revealed that the patient’s coagulation was normal, an endoscopic L5/S1 laminectomy and discectomy were performed. An arterial ultrasonic doppler was also used before surgery to exclude preoperative thrombus formation in the lower limbs. The endoscopic decompression proceeded via continuous irrigation with a flow rate of 150 mL/min. The surgeon was young and a novice, with no more than ten cases of experience in spinal endoscopic surgery. Hence, while performing the surgery, coping with a calcified herniated disk, which caused a small-sized dural tear resulting in CSF leakage, was challenging for him (Supplementary intraoperative Video S1: https://www.dropbox.com/s/joj0uos7vgac2aw/modified%20video.mp4?dl=0) (Accessed date: 1 January 2023). Subsequently, the surgery was extended for another 1 h by an experienced endoscopic surgeon to rectify the complication. Moreover, to acquire a clearer surgical view, the flow rates for the irrigation fluid were increased up to 250 mL/min. The total operative duration was 1.5 h.

**Figure 2:**
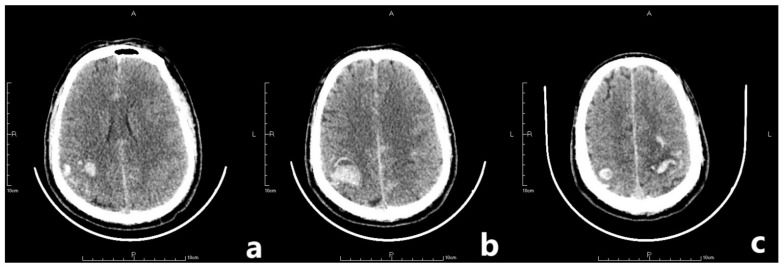
The letter R, A, P, L in the figures refers to Right, Anterior, Posterior and Left, respectively. Emergency CT scan showed scattered hemorrhages in bilateral frontoparietal lobes combined with subarachnoid hemorrhage and edema zones around some lesions (**a**–**c**).

**Figure 3:**
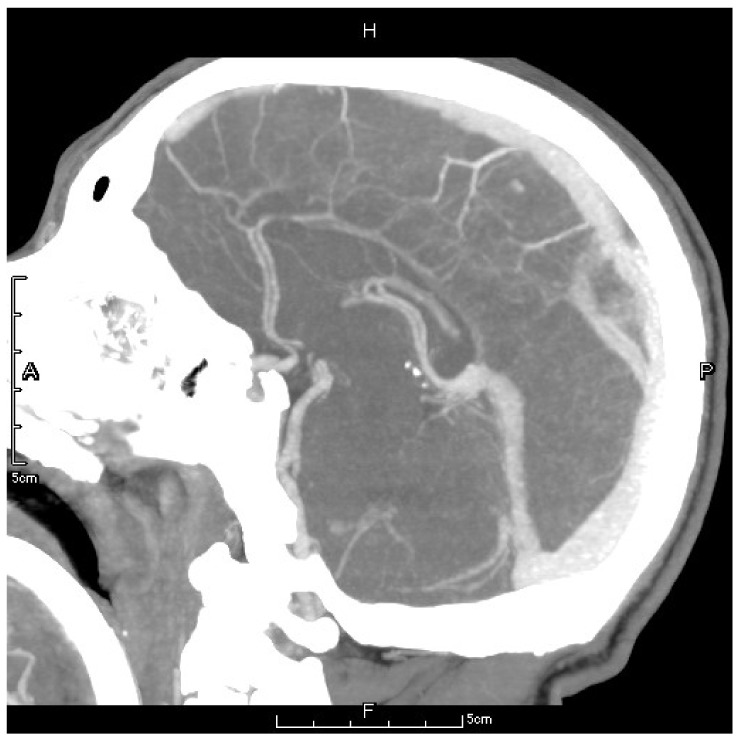
The letter F, A, P, H in the figures refers to Foot, Anterior, Posterior and Head, respectively. Preoperative CTV showed a local patchy filling defect shadow behind the superior sagittal sinus which should be considered as thrombus formation.

On the second postoperative day, the patient presented with idiopathic unconsciousness, limb twitching, and trismus. An emergency cranial CT scan demonstrated scattered hemorrhages in the bilateral frontoparietal lobes along with subarachnoid hemorrhages and edematous zones around some lesions (Figure 2 and Figure 3). Following an emergency consultation with the Department of Neurology and Neurosurgery, a diagnosis of venous sinus thrombosis combined with multi-focus cerebral hemorrhage was made. As per their orders, the patient underwent an emergency interventional intracranial angiography and thrombectomy.

### 2.3. Operational Procedure

**Figure 4:**
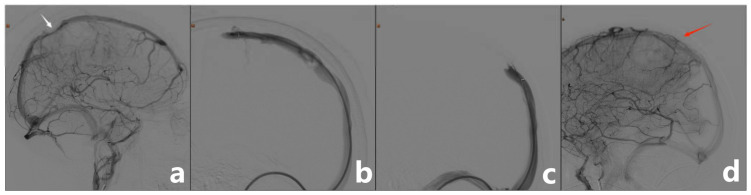
(**a**) Cerebral angiography showing filling defect of the superior sagittal sinus (white arrow). (**b**,**c**) The guidewire was delivered to the superior sagittal sinus thrombus and the stent was delivered along the microcatheter to the thrombus and released for thrombus retrieval. (**d**) Post-embolization imaging suggested complete recanalization of the superior sagittal sinus (red arrow).

Under general anesthesia and electrocardiogram monitoring, the patient was placed in the supine position and routine disinfection and draping of the surgical area were performed. The puncture needle was pierced into the right femoral artery using the modified Seldinger technique. Then, a 5F radial artery special-purpose puncture sheath was inserted. Through angiography, a 20 mm filling defect was observed at the superior sagittal sinus with slow blood velocity, which could be considered a thrombus formation at the superior sagittal sinus. Then, the right jugular vein was punctured using the modified Seldinger technique, and a 6F radial artery special-purpose puncture sheath was inserted. Next, the guidewire was delivered to the superior sagittal sinus thrombus, and the stent was delivered along the microcatheter to the thrombus and released for thrombus retrieval. The thrombus was subsequently extracted. Finally, post-embolization imaging suggested complete superior sagittal sinus recanalization (Figure 4).

### 2.4. Postoperative Management

**Figure 5:**
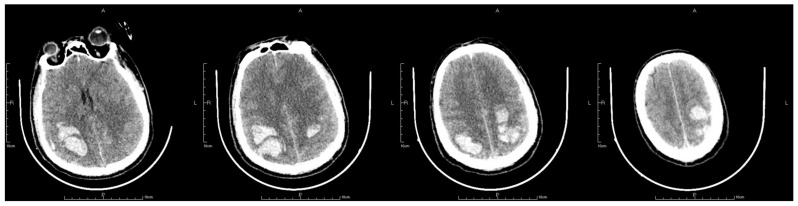
The letter R, A, P, L in the figures refers to Right, Anterior, Posterior and Left, respectively. CT scan after thrombectomy showed postoperative changing with expanded hemorrhage areas in bilateral frontoparietal lobes together with bilateral intraparenchymal hematoma in the frontoparietal lobes.

**Figure 6:**
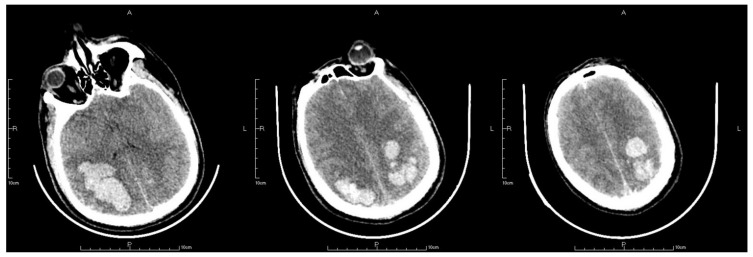
The letter R, A, P, L in the figures refers to Right, Anterior, Posterior and Left, respectively. Last CT before death showed hemorrhage areas on right frontoparietal lobes and further expansion of epencephalon.

After the surgery, routine postoperative management was employed. Mask inhalation was applied constantly with a flow rate of 5 L/min; 250 mL mannitol was used to reduce the intracranial pressure; 8 mg noradrenaline was constantly pumped in to maintain blood pressure within a normal range; and 50 μg sufentanil was also pumped in for sedation and pain relief. However, 2 h after the surgery, the patient suddenly presented with vomiting and idiopathic myosis with a diameter of only 1.5 mm, and did not react to light. An emergency CT scan revealing a postoperative change after the thrombectomy was obtained. The CT scan showed expanded hemorrhagic areas along with bilateral intraparenchymal hematomas in the frontoparietal lobes (Figure 5). Concurrently, the emergency arterial blood gas revealed the following: pH, 7.296; PCO_2,_ 32.6 mmHg; PO_2,_ 63.5 mmHg; and cHCO_3_, 15.5 mmol/L. Emergency tracheal intubation with an invasive ventilator was implemented to ensure that the airway was unobstructed. Mannitol was applied again to enhance intracranial pressure reduction. Another cerebral CT scan was also obtained on the first postoperative day, which demonstrated that the hemorrhagic areas on the right frontoparietal lobes and epencephalon had further expanded (Figure 6). On the second postoperative day, the patient’s autonomous respiration and heartbeat ceased concomitantly with corectasis. The emergency electrocardiogram depicted an equipotential line, following which the patient was pronounced dead.

## 3. Discussion

PIH is considered a rare but horrible postoperative spinal complication. However, its causative factor has never been confirmed by previous researchers. Paul E Kaloostian et al. retrospectively reviewed eight patients treated for over 16 years and diagnosed with intracranial hemorrhage after spinal surgery. They attributed the cause of PIH to CSF leakage and the use of drains postoperatively [11]. Hassan Allouch et al. conducted retrospective research of 10 patients with PIH 6 y after spinal surgeries. According to their research, the element leading to PIH was also related to CSF leakage. The authors underlined that intracranial bleeding must be considered in every patient with unexplained neurological deterioration after spinal surgery and should be ruled out via cranial imaging [12]. Mahmoud Reza Khalatbari et al. reported a series of PIH cases. They pointed out that PIH might be asymptomatic or manifest with an intense headache in the early stages at any time during the first week after surgery [4]. Ryan C Hofler et al. reported a case study of a patient without durotomy who suffered PIH after lumbar surgery. They concluded that hypertension was a risk factor for intracranial hemorrhage. In patients with unusual presentations of PIH, it is important to plan a careful workup and management [13]. The pathway of the pathology used to be controversial. With the increasing number of studies, intracranial hypotension has been given great importance in the causation of PIH [14,15,16,17,18]. CSF volume loss is considered to result in intracranial pressure reduction with subsequent enlargement of the dural venous sinuses. This predisposes the patient to a subdural hematoma [18,19]. Caudal brain displacement due to the pressure decrease could further increase the risk of venous tears on already enlarged venous sinuses [20].

Thus far, PIH occurring after spinal endoscopic surgery has never been reported. In our conventional concept, minimally invasive spinal surgery, such as endoscopic spinal surgery, which is now considered a state-of-the-art technique, should technically prevent this severe complication to a large extent [21,22,23,24]. However, a few key points that seem insignificant might have long been ignored. Additionally, the factors resulting in PIH in this patient were not the same as those previously analyzed in the literature. In this patient, the dural tear occurred because of inappropriate manipulation carried out by the inexperienced surgeon. It induced a subsequent long surgical time, and an experienced surgeon intervened to get the surgery back on track. However, unlike traditional open surgery or other minimally invasive surgical spine techniques (for instance, tubular retractor), spine endoscopy highlights the merit of constant irrigation [25]. After durotomy, the surgical view becomes blurred. To cope with this, the irrigation saline is elevated to increase the hydraulic pressure, probably causing intracranial hypertension [26]. This might increase the risk of intracranial hemorrhage formation, which is a chain reaction that occurs due to the increment in blood volume, leading to a reduction in the blood flow velocity [27,28,29]. As feedback, the intracranial hemorrhage formation would deteriorate intracranial hypertension [30], thus leading to the occurrence of PIH. 

Endoscopic spinal surgery is an outstanding minimally invasive technique. However, durotomy still cannot not be averted [31,32,33]. Its incidence was once reported as 0.54% [34]. To prevent the occurrence of PIH, surgeons should keep a few principal concepts in mind. Firstly, it is not recommended to elevate the irrigation saline beyond 200 mL/min [26]. As in several practices, it is impossible for durotomy occurrence to be noticed every time [14,35]. In this regard, too much hydraulic pressure could perhaps be a potential risk for PIH [31]. Secondly, once durotomy occurs, alternative measures should be taken. Meaningful work has already been conducted to codify the workflow for dealing with durotomy [32,36,37]. Its management, mainly regarding whether the small size of the durotomy should be repaired or not, seems controversial. However, a consensus has been reached about its management, stating that once the dural tear is observed, one of the following three choices should be made: finalizing the procedure at once, repairing under endoscopy, or switching to open repair. The original procedure should not be continued and the dural tear should be left alone. Otherwise, continuous irrigation would deteriorate the situation and result in a calamitous outcome. PIH could probably be effectively prevented in this way. However, further research should be conducted to gain more clarity. 

## 4. Conclusions

PIH after spinal endoscopic surgery is a rare but horrible complication. Several factors could lead to PIH. However, in this patient, PIH might be attributed to the long duration of surgery along with CSF leakage. Great attention should be attached to this issue while performing endoscopic spinal procedures due to constant irrigation. This study aimed to highlight the issue of PIH after spinal endoscopic surgery by presenting a case report of a patient who died despite successful endoscopic spinal surgery.

## Data Availability

Data sharing is not applicable to this article as no datasets were generated or analyzed during the study.

## Supplementary Materials

The following supporting information can be downloaded at: https://www.mdpi.com/article/10.3390/jpm13020196/s1, Supplementary Video S1: Video recorded intraoperatively showing a durotomy observed under endoscope.
