# Supplementary material for: Postoperative Intracranial Hemorrhage after an Endoscopic L5-S1 Laminectomy and Discectomy: A Case Report and Literature Review
Source: J Pers Med. 2023 Jan 22;13(2):196. doi: 10.3390/jpm13020196 (PMC9958620; doi:10.3390/jpm13020196)
Supplement: Supplementary file 1 [file jpm-13-00196-s001.zip › CARE-checklist-English-2013.pdf]

CARE case report guidelines

CARE Checklist of information to include when writing a case report

(cc) BY-NC-ND

Topic	Item	Checklist item description	Reported on Line
Title	1	The diagnosis or intervention of primary focus followed by the words "case report"	1-3
Key Words	2	2 to 5 key words that identify diagnoses or interventions in this case report, including "case report"	28-30
Abstract (no references)	3a	Introduction: What is unique about this case and what does it add to the scientific literature?	4-26
	3b	Main symptoms and/or important clinical findings	10-19
	3c	The main diagnoses, therapeutic interventions, and outcomes	10-19
	3d	Conclusion—What is the main "take-away" lesson(s) from this case?	20-26
Introduction	4	One or two paragraphs summarizing why this case is unique (**may include references**)	32-40
Patient Information	5a	De-identified patient specific information.	-
	5b	Primary concerns and symptoms of the patient.	32-40
	5c	Medical, family, and psycho-social history including relevant genetic information	-
	5d	Relevant past interventions with outcomes	-
Clinical Findings	6	Describe significant physical examination (PE) and important clinical findings.	43-64
Timeline	7	Historical and current information from this episode of care organized as a timeline	43-64
Diagnostic Assessment	8a	Diagnostic testing (such as PE, laboratory testing, imaging, surveys).	43-64
	8b	Diagnostic challenges (such as access to testing, financial, or cultural)	-
	8c	Diagnosis (including other diagnoses considered)	-
	8d	Prognosis (such as staging in oncology) where applicable	-
Therapeutic Intervention	9a	Types of therapeutic intervention (such as pharmacologic, surgical, preventive, self-care)	66-100
	9b	Administration of therapeutic intervention (such as dosage, strength, duration)	66-100
	9c	Changes in therapeutic intervention (with rationale)	-
Follow-up and Outcomes	10a	Clinician and patient-assessed outcomes (if available)	66-100
	10b	Important follow-up diagnostic and other test results	66-100
	10c	Intervention adherence and tolerability (How was this assessed?)	-
	10d	Adverse and unanticipated events	-
Discussion	11a	A scientific discussion of the strengths AND limitations associated with this case report	102-160
	11b	Discussion of the relevant medical literature **with references**.	102-160
	11c	The scientific rationale for any conclusions (including assessment of possible causes)	102-160
	11d	The primary "take-away" lessons of this case report (without references) in a one paragraph conclusion	102-160
Patient Perspective	12	The patient should share their perspective in one to two paragraphs on the treatment(s) they received	66-100
Informed Consent	13	Did the patient give informed consent? Please provide if requested	Yes ☑ No ☐
